# High-Sensitivity Intrinsic Optical Signal Imaging Through Flexible, Low-Cost Adaptations of an Upright Microscope

**DOI:** 10.1523/ENEURO.0046-23.2023

**Published:** 2023-08-04

**Authors:** Brenda Vasquez, Baruc Campos, Ashley Cao, Aye Theint Theint, William Zeiger

**Affiliations:** Department of Neurology, David Geffen School of Medicine, University of California, Los Angeles, Los Angeles, California 90095

**Keywords:** cortical plasticity, *in vivo* imaging, intrinsic signal optical imaging, somatosensory cortex

## Abstract

Intrinsic optical signal imaging (IOSI) is a staple technique in modern neuroscience. Pioneered >30 years ago, IOSI allows macroscopic mapping of neuronal activity throughout the cortex. The technique has been used to study sensory processing and experience-dependent plasticity, and is often used as an adjunctive procedure to localize cortical areas for subsequent targeting by other imaging or physiology techniques. Despite the ubiquity of IOSI in neuroscience, there are few commercially available turn-key IOSI systems. As a result, investigators have typically resorted to building their own imaging systems. Over the years, simplified systems built either as dedicated rigs or incorporated into existing microscope platforms have been developed. Here we present a straightforward set of adaptations that can be applied to any standard upright microscope, using readily available, inexpensive, commercial parts for illumination, optics, and signal detection, that enables high-sensitivity IOSI. Using these adaptations, we are able to readily map sensory-evoked signals across the somatosensory and visual cortex, including single-whisker barrel cortical activity maps in mice. We show that these IOSI maps are highly reproducible across animals and can be used to study plasticity mechanisms in the somatosensory cortex. We also provide open-source applications to control illumination and analyze raw data to generate activity maps. We anticipate that these resources will be useful for neuroscience investigators looking to add IOSI capabilities to an existing microscope in the laboratory on a budget.

## Significance Statement

Intrinsic optical signal imaging allows macroscopic detection of cortical activity. It has become a staple technique for modern neuroscience, yet there are few commercially available systems. Here we describe low-cost adaptations and open-source tools that enable the adaptation of an existing upright microscope to perform high-sensitivity intrinsic signal imaging experiments and basic data analysis.

## Introduction

Intrinsic optical signal imaging (IOSI) is a macroscopic imaging technique that can indirectly map neuronal activity by measuring changes in intrinsic signals (Frostig and Chen-Bee, 2009). Pioneered in the 1980s (Grinvald et al., 1986), IOSI has since become a staple technique in neuroscience. IOSI has been used to uncover the functional architecture of the brain, including the retinotopic (Crair et al., 1997; Schuett et al., 2002), somatotopic (Masino et al., 1993; Narayan et al., 1994), tonotopic (Bakin et al., 1996; Kalatsky et al., 2005), and odorant (Rubin and Katz, 1999) mapping of the cortex. It has also been critical for studies of cortical plasticity (Prakash et al., 1996; Trachtenberg et al., 2000; Polley et al., 1999, 2004; Dubroff et al., 2005; Lehmann and Löwel, 2008; Drew and Feldman, 2009; Smith and Trachtenberg, 2010; Kalogeraki et al., 2017) and to study the pathogenesis of diseases such as migraines (Ba et al., 2002), seizures (Chen et al., 2000), stroke (Clarkson et al., 2013; Harrison et al., 2013; Johnston et al., 2013), and neurodevelopmental disorders (Arnett et al., 2014; He et al., 2019). From a practical standpoint, IOSI can be used to localize cortical areas for subsequent interventions like viral vector injections (Augustinaite and Kuhn, 2021), two-photon imaging (He et al., 2017; de Vries et al., 2020), *in vivo* electrophysiology (Nsiangani et al., 2022), or focal lesions (Zeiger et al., 2021).

To conduct IOSI, one needs a light source to illuminate the cortex, optics to collect reflected light, and a detector (Zepeda et al., 2004; Frostig and Chen-Bee, 2009). Many different approaches to assembling these basic hardware components have been described, with most custom built by individual laboratories as there are few complete commercial IOSI setups available. Early setups for IOSI evolved from traditional upright microscopes with arrays of photodetectors (Grinvald, 1985; Grinvald et al., 1986) to a tandem-lens macroscope configuration with sensitive charge-coupled device (CCD) cameras (Ratzlaff and Grinvald, 1991). Illumination has shifted from broadband light sources with emission filters (Ratzlaff and Grinvald, 1991; Frostig et al., 1995) to simpler wavelength-specific light-emitting diodes (LEDs; Salzberg et al., 2005; Harrison et al., 2009; Augustinaite and Kuhn, 2021). Here, we incorporate aspects of prior IOSI designs and provide updated protocols that enable the addition of IOSI capabilities to an existing upright microscope. We use an inexpensive, commercially available LED ring light controlled by an Arduino microcontroller for illumination, a basic microscope objective for light collection, and mid-range cameras for detection to achieve high-sensitivity IOSI. In addition, we provide simple, easy-to-use MATLAB-based applications for illumination control and basic image analysis. This IOSI setup enables one to incorporate this versatile technique into the laboratory in a matter of hours for a very low budget. We anticipate it will be particularly useful for researchers performing IOSI in conjunction with other techniques requiring microscopy, such as two-photon *in vivo* imaging.

## Materials and Methods

### Experimental animals

All experiments followed the US National Institutes of Health guidelines for animal research, under an animal use protocol approved by the University of California, Los Angeles, Animal Research Committee. Male and female mice were used, beginning at 7–10 weeks of age at the time of cranial window surgery. All animals were housed in a vivarium with a 12 h light/dark cycle. For these experiments, we used one wild-type C57BL/6J mouse, three hemizygous transgenic Ai162D mice [Ai162(TIT2L-GC6s-ICL-tTA2)-D, Jax line 031562; Daigle et al., 2018], three homozygous and seven heterozygous PV-Cre mice [B6.129P2-Pvalbtm1(cre)Arbr/J; catalog #017320, The Jackson Laboratory; Hippenmeyer et al., 2005], two PV-Cre:Ai162 double-transgenic mice, and one Thy1*-*jRGECO1a transgenic mouse (catalog #030526, The Jackson Laboratory; Dana et al., 2018). All transgenic lines were maintained on a C57BL/6J background.

### Cranial window surgery

Implantation of chronic glass cranial windows was performed according to previously published protocols (Mostany and Portera-Cailliau, 2008; Holtmaat et al., 2009). Mice were deeply anesthetized using 5% isoflurane followed by maintenance with 1.5–2% isoflurane. The scalp was removed, and the periosteum was cleaned away by gentle scraping. An ∼4-mm-diameter circular craniotomy, centered ∼3 mm lateral to the midline and ∼2 mm caudal to bregma was made using a pneumatic dental drill with a FG one-quarter drill bit (Midwest Dental) over the primary somatosensory cortex, including the barrel field (S1BF), forelimb (S1FL), and hindlimb (S1HL) areas. For primary visual cortex (V1), the craniotomy location was shifted ∼1.5 mm caudal. The craniotomy was sealed using either a single 5 mm #1 sterile glass coverslip (Harvard Apparatus) or a 4 mm coverslip glued to a 5 mm coverslip using an optical adhesive (catalog #71, Norland Products), and was glued to the skull with cyanoacrylate glue (Krazy Glue) followed by dental acrylic (OrthoJet, Lang Dental). A small stainless steel headbar was placed rostral to the cranial window and embedded in dental acrylic to allow subsequent fixation of the mouse onto the microscope stage. Carprofen (5 mg/kg, i.p.; Zoetis) and dexamethasone (0.2 mg/kg, i.p.; Vet One) were provided for pain relief and mitigation of edema on the day of surgery and daily for the next 48 h. Mice were allowed to recover from the surgery for 3 weeks before the first imaging session. For thinned skull imaging, an ∼3-mm-diameter circle over S1BF was thinned until transparency using a pneumatic dental drill with a FG one-quarter drill bit (Midwest Dental), followed by headbar implantation and acute IOSI.

### IOSI hardware/software setup

We adapted an upright two-photon microscope (Bergamo II, Thorlabs) to perform intrinsic signal imaging. A 4× air-immersion objective [0.2 numerical aperture (NA); CFI Plan Apochromat Lambda, Nikon] was threaded into the microscope objective holder, and a ring of 16 LEDs with integrated drivers (NeoPixel Ring #1463, Adafruit) was affixed to the objective using a custom 3D-printed holder. The ring LED illumination was controlled using an Arduino microcontroller (Uno Rev3) and a custom-written MATLAB (MathWorks) application. Full details on how to install and set up the ring LED illumination, including files for 3D printing and MATLAB code can be found on github (https://github.com/zeigerlab/Intrinsic-Signal-Imaging). Light from the objective was transmitted directly to a camera tube and camera, either a 1× camera tube (catalog #WFA4100, Thorlabs) coupled to an 8 megapixel CCD camera (catalog #8051M-USB, Thorlabs; pixels, 3296 × 2472; pixel size, 5.5 × 5.5 μm; 14 bit depth; charge capacity, 20,000 e^–^) or a 0.5× camera tube (model WFA4102, Thorlabs) coupled to a 12.3 megapixel complementary metal-oxide semiconductor (CMOS) camera (pixels, 4096 × 3000; pixel size, 3.45 × 3.45 μm; 12 bit depth; charge capacity, ≥10,650 e^–^; model CS126MU, Thorlabs). A 5 V transistor–transistor logic (TTL) pulse generated 1 s prior to the onset of stimuli was used to trigger camera acquisition (model TSI-IOBOB, Thorlabs).

### IOSI acquisition

Animals were sedated with chlorprothixene (∼3 mg/kg, i.p.), lightly anesthetized with ∼0.5–0.7% isoflurane and head fixed below the microscope. The cortical surface was illuminated by green light (525 nm) to visualize and capture an image of the superficial vasculature. The microscope was then focused 300 μm below the cortical surface, and a red light (625 nm) was used to record intrinsic signals, with frames collected at 10 Hz starting 1 s before and up to 3 s after stimulation onset. For one mouse, we also performed IOSI using a frame acquisition rate of 30 Hz. Thirty trials with interstimulus intervals of 20 s were conducted for each imaging session. Paw and whisker stimuli (10 or 100 Hz sine wave, 1.5 s long) were generated in MATLAB, with output via a multifunction input/output device (catalog #BNC 2090a and #PCIe-6363, National Instruments) to a voltage amplifier (catalog #PD200-V100,100, Micromechatronics), and delivered using a glass capillary affixed to a piezoelectric bending actuator (Python PBA6014-5H200, Bimitech). Visual stimuli were generated using PsychoPy (Peirce et al., 2019) and consisted of drifting sinusoidal gratings (spatial frequency, 0.04 cycles/°; speed, 2 cycles/s) at orientations of 0°, 45°, 90°, and 135° (each displayed for 0.375 s for a total stimulus duration of 1.5 s) followed by a black screen during the interstimulus interval. Images were acquired with 100 ms exposure time at 10 frames/s, for a total of 4 s/trial.

### Quantification of evoked signals

Images were first Gaussian filtered using the *imgaussfilt* function in MATLAB, with the default kernel size of 2 * ceil(2 * sigma) + 1, with sigma = 0.5. Images were then spatially downsampled by a factor of four. For each trial, an average baseline reflectance image was created by calculating the mean across 0.9 s of images (nine frames) prior to stimulus onset. Poststimulus reflectance images were temporally averaged across 0.3 s bins for 1.5 s total, starting 0.5 s after stimulus onset, yielding five poststimulus images per trial. Change in reflectance values (Δ*R*/*R*) were then calculated by subtracting the average baseline reflectance from each poststimulus image and dividing the result by the average baseline reflectance. These Δ*R*/*R* values were then averaged across all 30 trials and finally summed across the five poststimulus images to yield a single total stimulus-evoked Δ*R*/*R* image. A circular mask corresponding to the area of the cranial window (based on the vasculature image) was fit to the stimulus evoked Δ*R*/*R* image such that pixels outside the cranial window were set to 0. Binary images were then created either by thresholding Δ*R*/*R* values using a percentage of the maximum signal intensity (for larger maps; e.g., S1BF or V1), or by first calculating *z* scores of the obtained Δ*R*/*R* values and then thresholding values below a *z* score of –3 (for smaller maps; e.g., single whisker, S1FL, or S1HL). Binarized images were then pseudocolored and overlaid onto images of the vasculature. To obtain group-averaged images and map displacements, binarized maps of the S1BF, S1FL, and S1HL were fit with an ellipse using the “Analyze Particles” function in Fiji/ImageJ (Schindelin et al., 2012). The center of each ellipse was determined, and stimulus-evoked Δ*R*/*R* images from each mouse were aligned using the center of the S1BF ellipse, overlaid, and averaged. Displacement of S1FL and S1HL relative to S1BF were then calculated for each mouse and compared using a one-way multivariate ANOVA (MANOVA). To quantify the map area for single whisker-evoked maps, the medfilt2 function in MATLAB was used to apply a median filter with a 3 × 3 pixel neighborhood size to binarized maps to remove noise, and the area of thresholded pixels was calculated. Prestimulus and post-whisker trimming map sizes were compared using a paired-sample, two-tailed *t* test. All values listed or plotted are mean ± SEM, unless otherwise specified.

### Whisker trimming

Animals were anesthetized with isoflurane (5% for induction, 1.5–2% for maintenance), and all whiskers on the right side of the snout, except those undergoing stimulation (B1, C1, and/or D1), were trimmed using a fine scissors to a length of ∼5 mm immediately prior to imaging. For the chronic whisker-trimming experiment, all whiskers on the right side of the face except C1 were trimmed flush with the vibrissal pad immediately following baseline imaging and retrimmed as needed to remove any whisker regrowth, approximately three times weekly, for a total of 3 weeks.

### Data availability

The code/software described in the article is freely available online at https://github.com/zeigerlab/Intrinsic-Signal-Imaging. The code is available in Extended Data 1.

10.1523/ENEURO.0046-23.2023.ed1 Extended Data 1 Extended data includes several additional files. “*ISI Parts List.xlsx*” is an excel spreadsheet listing all materials required to implement the IOSI adaptations described in this manuscript. “*IOSI_RingLED_IlluminationBuild.docx*” is a word document providing step-by-step instructions for assembling and setting up ring LED illumination. “*Ring LED Holder-2.stl*” is a design file to 3D print the mount for the ring LED. “*NeopixelControl.mlapp*” is a MATLAB application for controlling ring LED illumination. “*iosgui.mlapp*” is a MATLAB application for processing IOSI data. “*IOSGUI_ImageAnalysis.m*” is a companion script for processing IOSI data, used by “iosgui.mlapp”. “*IOSGUI_Instructions.docx*” is an instruction manual for using the iosgui application for processing IOSI data. Download Extended Data 1, ZIP file.

## Results

**Figure 1.**
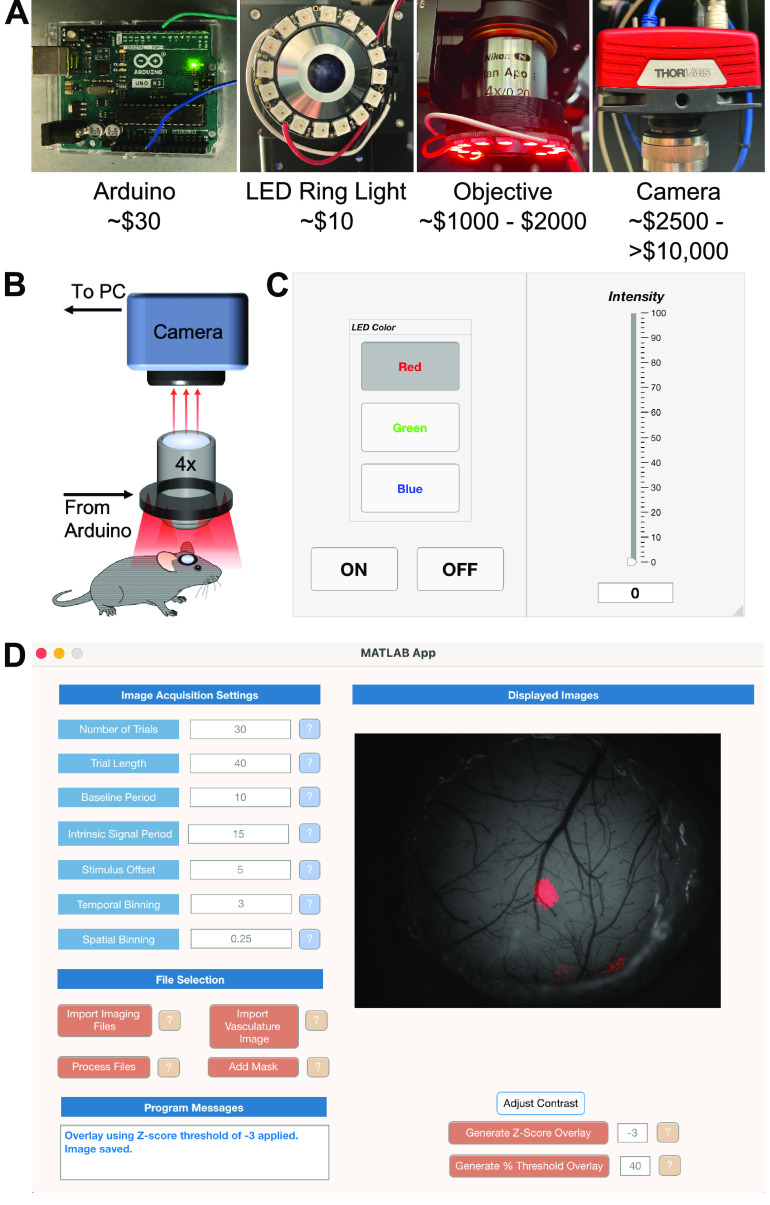
Hardware and software adaptations to enable IOSI on an existing upright microscope. ***A***, Hardware necessary to achieve IOSI includes (from left to right) an Arduino microcontroller connection to an LED ring light for illumination, an objective for light collection, and a camera to record reflected light. ***B***, Schematic of the hardware adaptations and basic connections. ***C***, Image of the application for illumination control. Users may set the illumination wavelength and intensity. ***D***, Image of the application for basic image analysis. Users may input settings used for image acquisition and identify acquired images. The application will then process images, calculate Δ*R*/*R* values, and display a scaled image in the application window. Thresholding can then be done within the application, using either an absolute percentage of the maximum Δ*R*/*R* value or using *z* scores of Δ*R*/*R* values, and a binarized map can then be overlaid onto an image of the vasculature for localization of signals.

To adapt an existing upright microscope for IOSI, we designed a simple, easy to implement system for sample illumination and image acquisition (Fig. 1*A*). The brain surface is illuminated using an array of LEDs, and reflected light is collected through a 4× microscope objective and transmitted to a scientific camera without any intervening filters (Fig. 1*B*). For sample illumination, we used a prefabricated LED ring light controlled by an Arduino microcontroller (see Materials and Methods). These LEDs have integrated drivers and require only a basic 5 V AC/DC power adapter. The ring light is slipped onto a 4× microscope objective using a 3D-printed mount and provides even sample illumination at wavelengths of ∼470, ∼525, or ∼625 nm. Control of the illumination wavelength and light intensity is achieved with a MATLAB-based application (Fig. 1*C*). Camera acquisition can then be synchronized to stimuli of interest via a TTL pulse from the imaging computer. After image acquisition, images are processed using a MATLAB-based application (Fig. 1*D*) to generate scaled change in reflectance (Δ*R*/*R*) images or are further thresholded and overlaid onto an image of brain vasculature for the localization of signals. The image analysis application is flexible with customizable inputs for specific acquisition settings, including imaging frame rate, number of trials, baseline and stimulus duration, and temporal binning, among others (Fig. 1*D*). The entire system can be set up on an upright microscope in a few hours for a cost of <$5000, or <$50 if the microscope already has an appropriate objective and camera. Complete installation instructions and design files are freely available online (https://github.com/zeigerlab/Intrinsic-Signal-Imaging).

**Figure 2.**
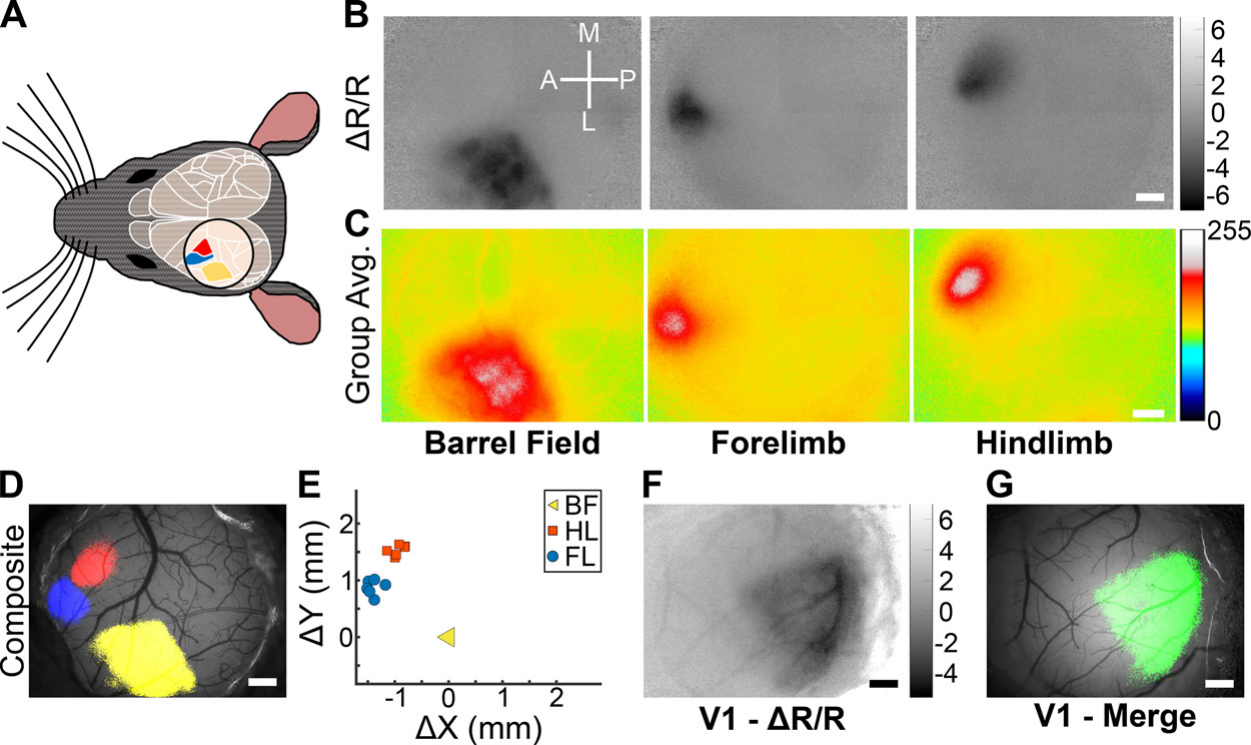
Mapping-evoked signals across primary somatosensory and visual cortices. ***A***, Schematic of cranial window placement for mapping evoked signals in the primary somatosensory cortex. Approximate locations of major cortical regions are outlined in white (adapted from the Allen Mouse Brain Atlas Brain Explorer 2), with the S1FL (blue), S1HL (red), and S1BF (yellow) color coded. ***B***, Scaled Δ*R*/*R* images of IOSI from an individual mouse performed during vibrotactile stimulation of the contralateral whiskers, forelimb, or hindlimb. Scaled Δ*R*/*R* values are ×10^−4^. Scale bar, 0.5 mm. ***C***, Group-averaged images from six mice of IOSI performed during vibrotactile stimulation of the contralateral whiskers, forelimb, or hindlimb. Values are arbitrary units of 8 bit images from minimum (0) to maximum (255). Scale bar, 0.5 mm. ***D***, Merged sensory-evoked maps from ***B*** overlaid onto the cortical vasculature. Δ*R*/*R* images were *z* scored and thresholded for values less than –3, binarized, pseudocolored (S1FL, blue; S1HL, red; S1BF, yellow), then merged and overlaid. Scale bar, 0.5 mm. ***E***, Displacement of S1FL and S1HL map centers, relative to the S1BF map, from 6 individual mice. Map displacements for S1FL and S1HL were significantly different from one another (one-way MANOVA, *p* = 1.04 × 10^−5^). ***F***, Scaled Δ*R*/*R* images of IOSI from an individual mouse performed during passive viewing of drifting sinusoidal gratings by the contralateral eye. Scaled Δ*R*/*R* values are ×10^−3^. Scale bar, 0.5 mm. ***G***, Visual-evoked map from ***F*** overlaid onto the cortical vasculature. Scale bar, 0.5 mm.

IOSI is commonly used to localize areas of the mouse primary somatosensory cortex (S1). Using our setup, we performed IOSI through a chronic cranial window implanted over the left primary somatosensory cortex during vibrotactile stimulation of the contralateral whiskers, forelimb, or hindlimb (Fig. 2*A*). We were able to record robustly evoked signals corresponding to the barrel field (S1BF), forelimb (S1FL), or hindlimb (S1HL) in individual mice (Fig. 2*B*). We then aligned the S1BF signals across six different animals and found that the group-averaged signal for each sensory area was highly localized (Fig. 2*C*), demonstrating that the location of these maps is consistent and reproducible across animals. By thresholding and merging the S1BF, S1FL, and S1HL signals, we could clearly define distinct maps situated as expected anatomically (Fig. 2*A*,*D*; Lau et al., 2008). We next quantified the relative displacement of the center of the S1FL and S1HL maps relative to the center of the S1BF map in each of six mice (Fig. 2*E*). The displacements were tightly clustered by map (S1FL or S1HL), with the S1FL map displacements significantly distinct from the S1HL map displacements (MANOVA, *p* = 1.04 × 10^−5^). We also performed IOSI during presentation of drifting sinusoidal gratings to map the primary visual cortex and found robust visually evoked signals (Fig. 2*F*,*G*). Together, these results demonstrate that our IOSI setup is sufficiently sensitive to record evoked signals from several major cortical sensory areas.

**Figure 3.**
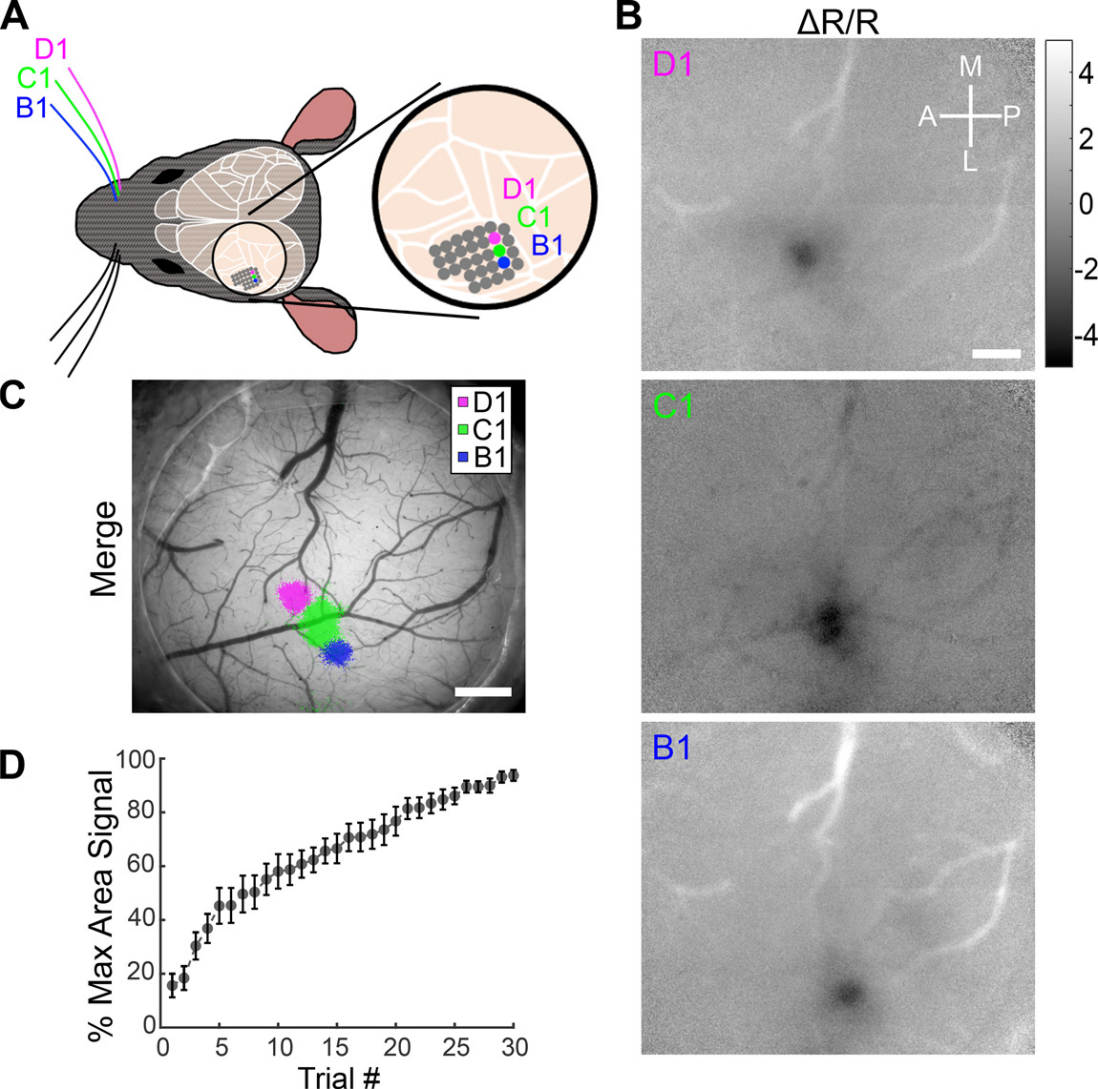
IOSI of single-whisker evoked cortical activity in the S1BF. ***A***, Schematic of stimulated whiskers (B1, C1, and D1) and somatotopic organization of the corresponding barrels in the S1BF. ***B***, Scaled Δ*R*/*R* images of IOSI from an individual mouse performed during vibrotactile stimulation of the contralateral B1, C1, and D1 whiskers. Scaled Δ*R*/*R* values are ×10^−3^. Scale bar, 0.5 mm. See Extended Data Figure 3-1 for scaled Δ*R*/*R* images of IOSI performed using higher frame rate acquisition (30 Hz) or acute thinned skull preparation. ***C***, Merged single-whisker maps from ***B*** overlaid onto the cortical vasculature. Δ*R*/*R* images were *z* scored and thresholded for values less than –3, binarized, pseudocolored, then merged and overlaid. Scale bar, 0.5 mm. ***D***, Average map area by IOSI trial, as a percentage of the maximum map area following all 30 trials, of 18 single-whisker representations in 6 mice.

**Figure 4.**
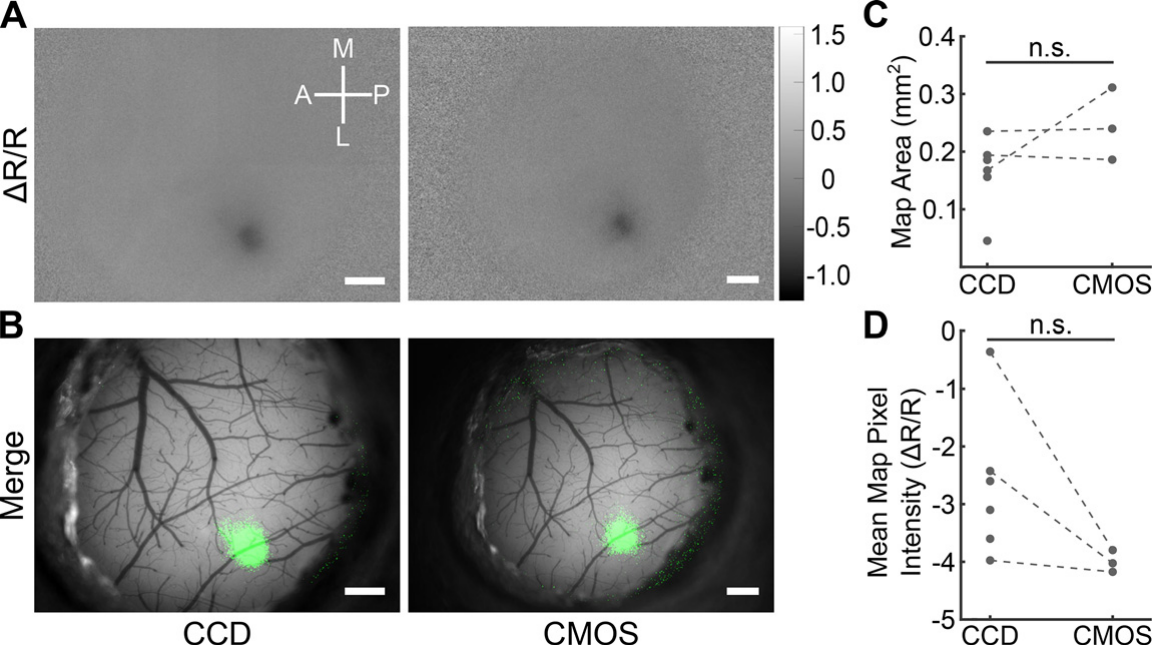
High-sensitivity IOSI can be achieved with CCD or CMOS cameras. ***A***, Scaled Δ*R*/*R* images of IOSI from an individual mouse performed during vibrotactile stimulation of the contralateral C1 whisker. Scaled Δ*R*/*R* values are ×10^−2^. Scale bar, 0.5 mm. ***B***, Merged single-whisker maps from ***A*** overlaid onto the cortical vasculature. Δ*R*/*R* images were *z* scored and thresholded for values less than –3, binarized, pseudocolored, then merged and overlaid. Scale bar, 0.5 mm. ***C***, Quantification of the C1 single-whisker map area from 6 mice imaged with the CCD camera and 3 mice imaged with the CMOS camera. Dotted lines indicate the same mouse imaged with both cameras, with imaging using the CMOS camera performed 6 d after the start of chronic whisker trimming (Fig. 5). Two-tailed *t* test, *p* = 0.13. ***D***, Quantification of C1 single-whisker map mean Δ*R*/*R* pixel intensity from the same mice as in ***C***. Δ*R*/*R* values are ×10^−3^. Two-tailed *t* test, *p* = 0.11.

We next tried to record evoked signals from smaller cortical areas. The mouse S1BF exhibits strong somatotopic organization, with sensory signals from individual whiskers predominantly encoded within single cortical columns, or barrels, that are ∼200–300 μm in diameter (Petersen, 2019). We performed IOSI during vibrotactile stimulation of single whiskers (B1, C1, of D1) and recorded robust single-whisker evoked signals (Fig. 3*A*,*B*). After thresholding and binarizing to obtain single-whisker maps, we merged the three individual single-whisker maps and found that these were clearly distinct and adjacently arranged according to the expected somatotopic organization of the S1BF (Fig. 3*C*). To quantify the minimum number of trials necessary to obtain reliable single-whisker maps, we calculated the cumulative change in reflectance values (Δ*R*/*R*) across each of the 30 trials performed in an individual IOSI session for 18 single-whisker maps (three whiskers in each of six mice). We then calculated the area of the evoked signal for each trial as a percentage of the maximum area obtained using all 30 trials (Fig. 3*D*). We found that the map area plateaued after 25–30 trials but was already >50% of the total map area after as few as 10 trials. Faster frame acquisition rates can improve signal-to-noise ratios for IOSI, especially with lower-sensitivity cameras. We found imaging at 10 Hz yielded a qualitatively similar single-whisker map compared with imaging at 30 Hz (Extended Data Fig. 3-1*A*). Single-whisker maps could also be readily obtained acutely through thinned-skull preparations (Extended Data Fig. 3-1*B*). Finally, we confirmed that we could generate qualitatively similar single-whisker maps to those obtained with a CCD camera using a more cost-efficient CMOS camera (Fig. 4*A*,*B*) and that there was no significant difference in average map size and mean map pixel intensity comparing IOSI performed with the CCD or CMOS camera (Fig. 4*C*,*D*). It is likely that the CCD camera could achieve higher signal-to-noise ratios compared with the CMOS camera by binning pixels on the CCD sensor, but we did not test that directly here. However, we can conclude that our IOSI setup can incorporate both CCD and CMOS cameras to produce reliable sensory-evoked maps even from signals generated by relatively small cortical areas.

10.1523/ENEURO.0046-23.2023.f3-1 Figure 3-1 IOSI of single whisker-evoked cortical activity in the S1BF using higher frame rates or an acute thinned skull cranial window. ***A***, Scaled Δ*R*/*R* images of IOSI from an individual mouse performed during vibrotactile stimulation of the contralateral C1 whisker, with imaging at 10 Hz (left) or 30 Hz (right). Scaled Δ*R*/*R* values are ×10^−3^. Scale bar, 0.5 mm. ***B***, Scaled Δ*R*/*R* images of IOSI through the acutely thinned skull from an individual mouse performed during vibrotactile stimulation of the contralateral D1 whisker. Scaled Δ*R*/*R* values are ×10^−3^. Scale bar, 0.5 mm. Download Figure 3-1, EPS file.

**Figure 5.**
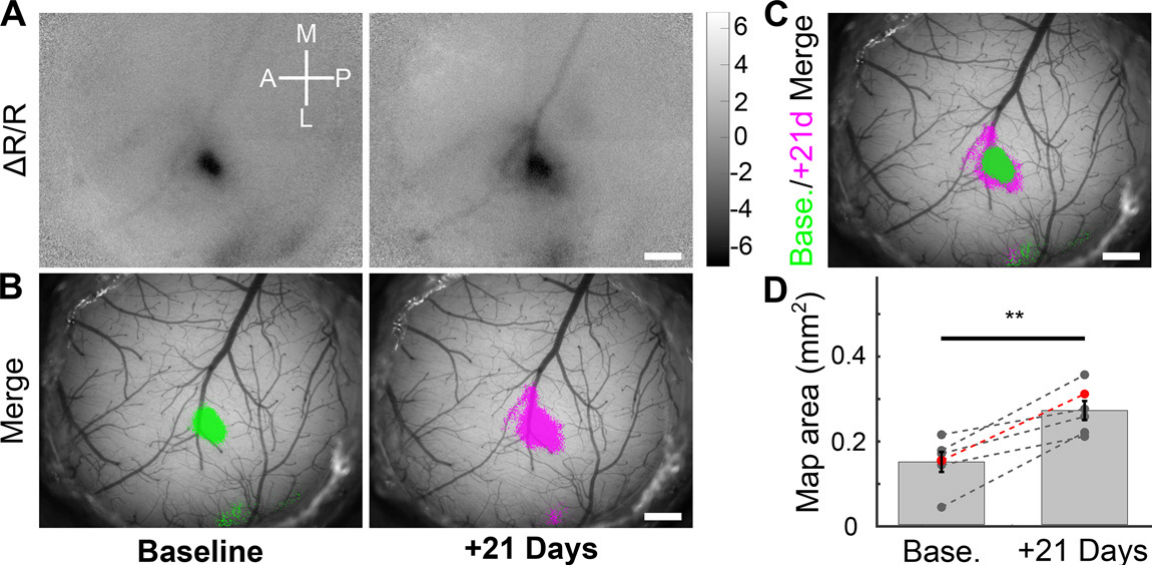
Longitudinal IOSI detects cortical map plasticity following whisker trimming. ***A***, Scaled Δ*R*/*R* images of IOSI from an individual mouse performed during vibrotactile stimulation of the contralateral C1 whisker at baseline before (left) and 21 d after whisker trimming sparing only the C1 whisker. Scaled Δ*R*/*R* values are ×10^−3^. Scale bar, 0.5 mm. ***B***, Merged single-whisker maps from ***A*** overlaid onto the cortical vasculature. Δ*R*/*R* images were *z* scored and thresholded for values less than –3, binarized, pseudocolored, then merged and overlaid. Scale bar, 0.5 mm. ***C***, Merged pre-whisker trimming and post-whisker trimming maps, aligned using vasculature images, showing expansion of the C1 whisker cortical map representation. Scale bar, 0.5 mm. ***D***, Quantification of change in C1 map area after whisker trimming from 6 mice. The dots in red represent the mouse depicted in ***A–C***. Paired-sample, two-tailed *t* test, **p = 0.003.

In addition to localizing signals, IOSI can be used to study cortical map plasticity. One of the most commonly used paradigms for inducing cortical map plasticity in rodents is whisker trimming. Following chronic trimming of whiskers, cortical map areas corresponding to the spared whisker expand, whereas those corresponding to trimmed whiskers shrink (Polley et al., 1999; Drew and Feldman, 2009; Gao et al., 2017). Therefore, we tested whether we could measure the plasticity of single-whisker maps in response to whisker trimming. Following baseline IOSI of C1 whisker evoked maps (Fig. 5*A*,*B*, left panels), we chronically trimmed all whiskers of the contralateral vibrissal pad except for C1. Three weeks later, we repeated IOSI during C1 whisker stimulation and quantified the area of the evoked C1 map (Fig. 5*A*,*B*, right panels). We merged the resulting pre-whisker trimming and post-whisker trimming maps and found that the C1 whisker map location was stable, but had increased in size, as expected, following chronic whisker trimming, sparing only the C1 whisker (Fig. 5*C*). To quantify this effect, we calculated the C1 whisker-evoked map area for six mice pre-trimming and post-trimming and found an ∼82% increase in map area after trimming (Fig. 5*D*; pretrimming = 0.16 ± 0.03 mm^2^; post-trimming = 0.30 ± 0.03 mm^2^; paired-sample, two-tailed *t* test, *p* = 0.003). Thus, our IOSI setup yields highly sensitive, quantitative cortical maps that can be used to both localize cortical signals as well as study fundamental processes such as cortical map plasticity.

## Discussion

Here we describe a set of simple adaptations that can be applied to an existing upright microscope to allow high-sensitivity IOSI. These adaptations make use of readily available commercial parts, can be implemented in an afternoon (or less), require no custom machining, and do not require any specialized skills in optics, electronics, circuit design, or programming. The entire setup can be implemented at extremely low cost: ∼$5,000 for an entire setup or <$50 if the microscope to be adapted is already equipped with a sufficient camera and objective. In addition, we provide simple MATLAB-based applications for controlling illumination and processing acquired images. Using this setup, we were able to record strong evoked signals from major areas of the mouse somatosensory cortex, including the S1BF, S1FL, S1HL, and V1. These maps show excellent signal-to-noise and are highly reproducible across mice. We were also able to record sensory-evoked signals from smaller cortical areas, consistently generating clear single-whisker evoked cortical maps from several adjacent whiskers that aligned with the expected somatotopy. Finally, we found that our IOSI setup could be used to quantitatively measure cortical map plasticity, detecting a significant increase in the cortical map area of a spared whisker after chronic whisker trimming.

The IOSI setup we describe here incorporates methods from previously described IOSI systems with updated, low-cost hardware. The first is hardware for sample illumination. Early IOSI setups used broadband light sources integrated into the microscope or macroscope housing, collimated and passed through excitation filters (Grinvald et al., 1986; Ratzlaff and Grinvald, 1991). Later iterations incorporated external light sources on flexible light guides to simplify construction (Frostig et al., 1995; Augustinaite and Kuhn, 2021; Nsiangani et al., 2022). More recently, it was recognized that LEDs could provide a stable, low-cost illumination source for IOSI (Salzberg et al., 2005; Harrison et al., 2009). We chose the LED ring light used here for the following several reasons: (1) it is commercially available at low cost; (2) it allows for three color illumination (including 525 nm for visualizing vasculature and 625 nm for IOSI); (3) it has integrated drivers and can be controlled with a basic Arduino microcontroller; (4) it is powered by a simple 5 V AC/DC power adapter plugged into a standard electrical outlet; and (5) it can be easily mounted using a simple 3D printed mount. Together, these features allow for stable, even illumination of samples that can be connected with just a few wires.

For collection optics, we used an off-the-shelf, commercial 4× plan apochromatic microscope objective. A tandem-lens macroscope is often used for light collection, given an excellent combination of field of view (FOV), working distance, and numerical aperture (Ratzlaff and Grinvald, 1991). However, microscope optics have advanced, and now even relatively inexpensive microscope objectives have sufficient numerical apertures (NA ∼0.1–0.2 compared with ∼0.4 for a tandem lens configuration). For cameras, we tested both a CCD and a CMOS scientific microscope camera. Since intrinsic signals are small, ∼0.01–0.1% of total reflected light, having a camera with sufficient sensitivity is essential (Frostig et al., 1995). The cameras we tested here had specifications (and costs) in the mid-range of available scientific cameras in the same class, and both were able to achieve qualitatively similar results mapping single-whisker evoked responses. Generally speaking, CCD cameras can achieve higher sensitivity and lower noise compared with CMOS cameras. CCD camera sensors can also perform pixel binning prior to signal readout, resulting in greater sensitivity and signal-to-noise ratio than can be achieved by CMOS cameras. For cameras with lower sensitivity, such as CMOS cameras, imaging more frames with shorter exposure times (i.e., increasing the frame rate) can improve the signal-to-noise ratio.

Our IOSI setup does have some limitations. We have not directly compared our system with a traditional tandem-lens macroscope. Such purpose-built IOSI rigs are cheaper for laboratories without an existing microscope available to adapt. The tandem-lens format also offers optical advantages in terms of fewer glasses for reflected light to travel through, potentially larger FOV, and better NA compared with microscope objectives. The LED ring light we used will not fit on larger objectives or tandem-lens macroscopes. However, the same LEDs are available as larger rings or strings of LEDs that can be shaped into custom forms. As such, the same illumination strategy can be adapted for a range of collection optics, including a tandem-lens configuration. Our illumination control application works well for constant single-wavelength illumination, but is not designed for multispectral imaging (Bouchard et al., 2009; White et al., 2011). It should be possible to achieve rapid wavelength switching synchronized to camera acquisition using the hardware we have described, but this would require additional customization. To date, we have used only two cameras in our experiments. We believe a range of modern CCD and CMOS cameras will likely function to achieve IOSI, given the mid-range technical specifications of the tested cameras. When comparing the CCD and CMOS cameras here we did not perform pixel binning, and it is likely that the CCD camera would outperform the CMOS camera if this were done. We refer readers to additional resources for choosing a particular camera for their imaging setup, which will depend on their budget and required sensitivity (Frostig and Chen-Bee, 2009; Harrison et al., 2009).

Regarding FOV, with a 4× objective, we achieved FOVs of ∼4.5 × 3.4 and ∼5.7 × 4.3 mm with the CCD and CMOS cameras, respectively. These FOV sizes can visualize the most common craniotomy sizes in mice but may not be sufficient for imaging larger cortical regions or animals. However, a larger FOV can be achieved by reducing the magnification on the microscope objective or camera tube lens, using a larger format camera sensor, and/or switching to a tandem-lens macroscope configuration. Our image-processing application allows users to adjust many settings specific to their particular imaging setup. However, we cannot guarantee functionality for more custom use cases. In addition, the application generates scaled Δ*R*/*R* images and maps overlaid onto the vasculature, but users may require more advanced image-processing tools in certain cases (e.g. retinotopic mapping of higher visual cortical areas). Finally, we have not provided detailed instructions here on generating stimuli to evoke cortical signals as we anticipate that these will vary significantly from laboratory to laboratory based on individual experimental paradigms.

We envision that these adaptations will be best suited to laboratories looking to add IOSI capabilities to an existing upright microscope in the laboratory. The primary advantages include low cost, ease of implementation, and a small footprint, given that a separate dedicated IOSI rig is not required. However, we anticipate that some of these adaptations or software tools may also be useful for more advanced users wanting to update components of existing rigs or incorporate these adaptations into custom-built stand-alone IOSI rigs.

## Synthesis

Reviewing Editor: Michael Michaelides, NIDA-NIH Decisions are customarily a result of the Reviewing Editor and the peer reviewers coming together and discussing their recommendations until a consensus is reached. When revisions are invited, a fact-based synthesis statement explaining their decision and outlining what is needed to prepare a revision will be listed below. The following reviewer(s) agreed to reveal their identity: Craig Brown.

Reviewer 1

To my surprise the authors seem to have ignored most of my comments and the current version of the manuscript seems almost identical to the original one with some minor changes in reaction to my review. The authors go again through the same rationale of how complicated, sophisticated a CCD camera system setup is, especially when it comes to some imaginary novice in the field. So here are the facts: CCD camera systems are not hard to get as the authors claim and they already come with user-friendly software. One can purchase new CCD systems from several companies (Andor, Photometrics, Princeton Instruments, to name a few). These camera systems come with their own advanced friendly software and can also be controlled by Matlab, Python or other free software packages. All what the ‘novice’ user must do then, is to mount the camera system and add a LED connected to a battery and a simple lens (a macroscope is only needed in few specialized cases and is easy to build even by any novice). So, in terms of ease, the CCD setup is way easier. The authors should also remember that their supposedly easy and cheap set up assumes that the novice user purchased and knows how to use or program in Matlab, ditto for Arduino, and that they have a 3D printer and know how to program it in case they want to change the provided files etc., all these take time and money to master and implement. Again, the authors discussion still has the awkward citing a 2009 paper from an author that didn’t hear that he can use a LED (Harris 2009), even though papers already at 2005 explained the use of LED (e.g., Salzberg b. m. et al. J. Neurosci. Methods 2005). Now the authors also claim that recent protocols don’t mention LEDs, but other sources of illumination, so what? Most researchers switched to LED illumination years ago without the need to follow protocols.

Instead of writing a simpleminded manuscript around ‘how to add IOSI to your existing microscope’, the introduction and discussion are busy in dealing with these unneeded, imaginary problems regarding traditional CCD systems and illumination. The manuscript should be rewritten by removing all these issues that don’t help, if not confuse, the reader.

There is another major subject in the paper that deals with the comparison of sCCDs and the newer style sCMOS cameras, where the authors claim that based on their results there is no difference between them. However, the way the comparison was performed was not a fair comparison. One of the main advantages of a sCCD camera systems over sCMOS is the ability to bin (2x2, 4x4, etc.) pixel values before the A/D conversion, which can nicely increase the signal/noise ratio and therefore can change quantification results (peak, area). However, the authors didn’t try to use the binning option and therefore misrepresented the ability of their CCD system. In addition, when comparing cameras, it would be nice to know some details about their cameras (pixel numbers, bit depth, depth of well for the CCD pixels, etc.). So, either the comparison is done again with binning (at least 2x2) or the authors should explain how their comparison is not optimal.

Reviewer 2

The authors addressed my previous comments. I think the paper is suitable for publication. Congratulations to the authors.

## Author Response

Reviewer 1

“To my surprise the authors seem to have ignored most of my comments and the current version of the manuscript seems almost identical to the original one with some minor changes in reaction to my review. The authors go again through the same rationale of how complicated, sophisticated a CCD camera system setup is, especially when it comes to some imaginary novice in the field. So here are the facts: CCD camera systems are not hard to get as the authors claim and they already come with user-friendly software. One can purchase new CCD systems from several companies (Andor, Photometrics, Princeton Instruments, to name a few). These camera systems come with their own advanced friendly software and can also be controlled by Matlab, Python or other free software packages. All what the ‘novice’ user must do then, is to mount the camera system and add a LED connected to a battery and a simple lens (a macroscope is only needed in few specialized cases and is easy to build even by any novice). So, in terms of ease, the CCD setup is way easier. The authors should also remember that their supposedly easy and cheap set up assumes that the novice user purchased and knows how to use or program in Matlab, ditto for Arduino, and that they have a 3D printer and know how to program it in case they want to change the provided files etc., all these take time and money to master and implement. Again, the authors discussion still has the awkward citing a 2009 paper from an author that didn’t hear that he can use a LED (Harris 2009), even though papers already at 2005 explained the use of LED (e.g., Salzberg b. m. et al. J. Neurosci. Methods 2005). Now the authors also claim that recent protocols don’t mention LEDs, but other sources of illumination, so what? Most researchers switched to LED illumination years ago without the need to follow protocols.

Instead of writing a simpleminded manuscript around ‘how to add IOSI to your existing microscope’, the introduction and discussion are busy in dealing with these unneeded, imaginary problems regarding traditional CCD systems and illumination. The manuscript should be rewritten by removing all these issues that don’t help, if not confuse, the reader.

There is another major subject in the paper that deals with the comparison of sCCDs and the newer style sCMOS cameras, where the authors claim that based on their results there is no difference between them. However, the way the comparison was performed was not a fair comparison. One of the main advantages of a sCCD camera systems over sCMOS is the ability to bin (2x2, 4x4, etc.) pixel values before the A/D conversion, which can nicely increase the signal/noise ratio and therefore can change quantification results (peak, area). However, the authors didn’t try to use the binning option and therefore misrepresented the ability of their CCD system. In addition, when comparing cameras, it would be nice to know some details about their cameras (pixel numbers, bit depth, depth of well for the CCD pixels, etc.). So, either the comparison is done again with binning (at least 2x2) or the authors should explain how their comparison is not optimal.”

Response to Reviewer 1:

We thank the Reviewer for their continued consideration of the manuscript. We have tried to revise the manuscript as suggested, framed around simple instructions for adding IOSI to an existing microscope, removing any reference to a novice user, removing discussion of perceived limitations of other traditional IOSI set-ups - including in the abstract, introduction, and discussion - and removing comparisons between our set-up and prior stand-alone set-ups. We have also added text and included the citation suggested by the reviewer in the introduction and discussion regarding earlier incorporations of LED illumination sources.

Regarding the quantitative comparison of the CCD and CMOS cameras, this was done to address a suggestion from Reviewer 2. We have added text in the results and in the discussion about the limitations of this comparison and the advantage of pixel binning to improve signal-to-noise ratio achievable by CCD cameras. We have added the requested camera specifications (pixel numbers, pixel size, bit depth, and well charge capacity) to the methods.

Reviewer 2

“The authors addressed my previous comments. I think the paper is suitable for publication. Congratulations to the authors.”

Response to Reviewer 2

We thank the Reviewer for their continued consideration of the manuscript.
