# Supplementary material for: High-Sensitivity Intrinsic Optical Signal Imaging Through Flexible, Low-Cost Adaptations of an Upright Microscope
Source: eNeuro. 2023 Aug 4;10(8):ENEURO.0046-23.2023. doi: 10.1523/ENEURO.0046-23.2023 (PMC10408783; doi:10.1523/ENEURO.0046-23.2023)
Supplement: Extended Data 1 — Extended data includes several additional files. “ISI Parts List.xlsx” is an excel spreadsheet listing all materials required to implement the IOSI adaptations described in this manuscript. “IOSI_RingLED_IlluminationBuild.docx” is a word document providing step-by-step instructions for assembling and setting up ring LED illumination. “Ring LED Holder-2.stl” is a design file to 3D print the mount for the ring LED. “NeopixelControl.mlapp” is a MATLAB application for controlling ring LED illumination. “iosgui.mlapp” is a MATLAB application for processing IOSI data. “IOSGUI_ImageAnalysis.m” is a companion script for processing IOSI data, used by “iosgui.mlapp”. “IOSGUI_Instructions.docx” is an instruction manual for using the iosgui application for processing IOSI data. Download Extended Data 1, ZIP file. [file enu-eN-OTM-0046-23-s02.zip › Extended Data/IOSGUI_Instructions.docx]

*
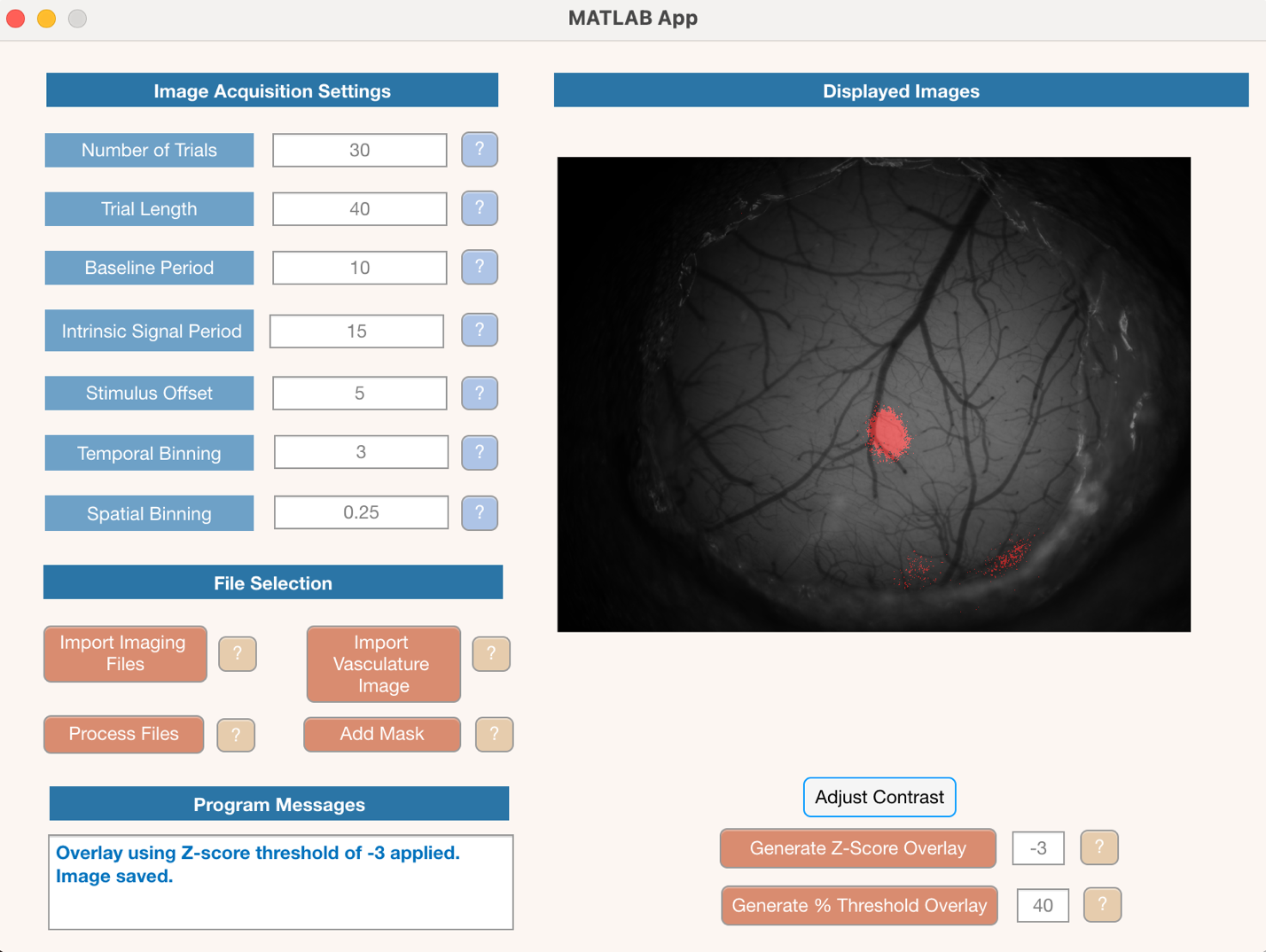
Quickstart Guide*

1. Open MATLAB and type “iosgui” to open the image processing application
2. Edit the fields in “Image Acquisition Settings” to match the settings used for acquiring IOSI images
3. Click “Import Imaging Files” and select a group of images (or an image stack) for processing
4. Click “Process Files” to start image processing and generate a scaled ΔR/R image
5. Click “Import Vasculature Image” to import an image of cortical vasculature taken contemporaneously with the IOSI images
6. *Optional* Click “Add Mask” and re-size the displayed ellipse to mask pixels outside the area of the ellipse
7. Click “Generate Z-score Overlay” or “Generate % Threshold Overlay” to generate an image with IOSI signals binarized and overlaid onto the cortical vasculature image

*Installation*

1. Clone the Intrinsic-Signal-Imaging repository from Github: [URL redacted for double-blind review]
   1. For detailed instructions, see <https://docs.github.com/en/repositories/creating-and-managing-repositories/cloning-a-repository?tool=desktop>
2. Add the folder containing the repository to your MATLAB path
   1. For detailed instructions, see: <https://www.mathworks.com/help/matlab/matlab_env/add-remove-or-reorder-folders-on-the-search-path.html>
3. Install the “Image Processing Toolbox” in MATLAB: https://www.mathworks.com/products/image.html

*Image Acquistion Settings*

- Number of Trials
  - Total number of trials to analyze. A single trial consists of a baseline period, a stimulus (whisker deflection, drifting visual grating, etc.), and a post-stimulus period. Typical IOSI experiments consist of usually 20-40 repeated trials. You may analyze a subset of recorded trials, but these must start at the first trial and run consecutively. If you have a different trial structure (for example, you only want to analyze every 3^rd^ trial), prior to analysis you will need to create a folder containing that particular sub-set of images, starting at the first trial to be analyzed, ordered and numbered consecutively.

**Figure 1. Required trial organization for analysis.** All (top) or a sub-set of trials (middle) may be selected for analysis, assuming trials are organized consecutively starting at the first trial acquired. Trial subsets that are non-consecutive or do not start at the first trial acquired (bottom) are not currently accommodated and require re-organization prior to image processing.

- Trial Length
  - The total number of frames (images) acquired for each individual trial. Include all acquired frames, even if some may not contribute to the analysis. For example, if you recorded for 4 seconds at 10 frames per second (or 10 Hz) for each trial, the trial length in frames will be 40.
- Baseline Period
  - The number of frames acquired before the stimulus of interest. For example, if the stimulus begins after 1 second of image acquisition at 10 Hz, the baseline period in frames will be 10. These images will be averaged on each trial to create a mean pre-stimulus reflectance image for the calculation of change in reflectance (ΔR/R) values.

**Figure 2. Structure of an individual trial.** Each trial is expected to consist of consecutively acquired frames comprising a pre-stimulus baseline period running from acquisition start to stimulus onset, an offset (optional) that allows for measurement of a desired phase of intrinsic signals, and the intrinsic signal period over which change in reflectance values will be calculated.


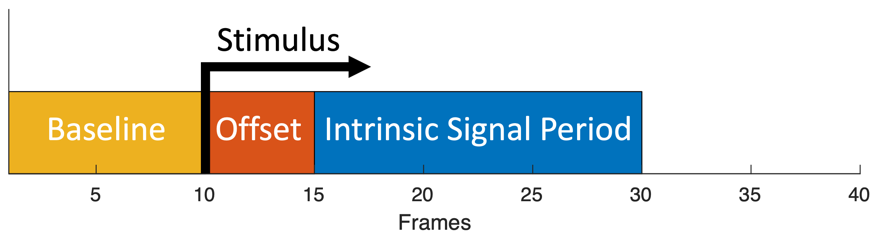


- Intrinsic Signal Period
  - The number of frames (images) acquired during the period over which you would like to calculate intrinsic signals. This period is typically 1-1.5 seconds, but may vary depending on the particular experiment and the specific intrinsic signal you are interested in quantifying. These images will be temporally binned (see below), normalized to the mean pre-stimulus image, and summed to calculate ΔR/R.
- Stimulus Offset
  - The number of frames (images) between the onset of the stimulus of interest and the beginning of the period over which you would like to calculate intrinsic signals. Intrinsic signals are triphasic and most experimenters quantify the first phase “initial dip”, which peaks ~1 second after stimulus onset and has the highest spatial correlation with neuronal activity. In this case, a stimulus offset of 0-0.5 seconds will probably work well for most experiments. However, longer offsets can be used if you are interested in more delayed phases of intrinsic signals. Of note, hemodynamic artifacts from local blood vessels become more significant in signals >1.5 seconds after stimulus onset.
    - For more details see “Frostig RD, Chen-Bee CH. Visualizing Adult Cortical Plasticity Using Intrinsic Signal Optical Imaging. In: Frostig RD, editor. In Vivo Optical Imaging of Brain Function. 2nd edition. Boca Raton (FL): CRC Press/Taylor & Francis; 2009. Chapter 9. Available from: <https://www.ncbi.nlm.nih.gov/books/NBK20227/>”
- Temporal Binning
  - The number of frames (images) over which to bin consecutively acquired images from the intrinsic signal period. Temporal binning can be helpful to reduce noise, increasing signal-to-noise ratio. Generally, higher acquisition rates can benefit from temporal binning. We have found temporal binning to 300-500 ms works well. For example, for images acquired at 10 Hz (~100 ms exposure) we use a temporal binning factor of 3 to achieve an ~300 ms temporal resolution.
- Spatial Binning
  - A multiplicative factor by which to spatially downsample images using a bilinear interpolation. For example, a 1000 x 1000 pixel image with a spatial binning factor of 0.25 will become a 250 x 250 pixel image. Spatial downsampling can improve signal to noise ratio, but at the expense of spatial resolution. We recommend trying a spatial binning factor of ~0.25-0.5 to start, but this may need to be optimized for your particular experimental set-up.

*File Selection*

- Import Imaging Files
  - Opens a window for file selection. Currently, the application can accept grayscale tiff image files in one of two formats:

1. Individual sequentially numbered files that share a common base name followed by an image number (such as “image_001.tif, image_002.tif, image_003.tif”).
   1. Image numbers should span trials – for example, if each trial is 40 frames long, the first frame of the second trial will be image 41, and so on.
   2. Hold “Control” or “Command” to select all images across all trials to be analyzed.
2. A multi-page tiff in which all images across all trials are sequentially acquired and stored together in one file.

- Process Files
  - This button will calculate intrinsic signals using the images selected previously and the settings defined in the input boxes in the “Image Acquisition Settings” section of the application.
  - A calculated ΔR/R image (scaled from minimum intensity to maximum intensity) will be displayed in the “Displayed Images” section of the application.
  - The scaled image and a MATLAB “.mat” file with raw data will be automatically saved in the directory containing the images selected for analysis. If scaled image and analysis MATLAB files already exist in the directory, a timestamp will be appended to the file names to prevent overwriting of data.
- Import Vasculature Image (Optional)
  - Opens a window for file selection. Choose a single grayscale tiff image of the cortical surface vasculature, typically acquired either immediately before or after an IOSI experiment.
  - The chosen vasculature image will be displayed in the “Displayed Images” section of the application.
  - In subsequent steps (see below) ΔR/R values can be binarized according to a threshold and overlaid onto this image to generate a map of intrinsic signals that can be localized according to the cortical vasculature.
- Add Mask (Optional)
  - This allows you to define areas of the acquired images to be excluded when generating overlay activity maps.
  - An ellipse will be displayed on the vasculature image in the “Displayed Images” section of the application.
  - Adjust and move the ellipse to fit the area of interest. The mask will automatically be updated each time the ellipse is adjusted or moved. Signals within the ellipse will be included and all signals outside of the ellipse will be excluded when generating overlay activity maps. This is useful, for example, for limiting signals to the area of a circular cranial window and excluding artifacts caused by surrounding hardware.

*Program Messages*

- Progress updates, error messages and warnings will be displayed in this area

*Displayed Images*

- Image Axes
  - Scaled ΔR/R images, imported vasculature images, and overlay images will be displayed on the axes in this area
- Adjust Contrast
  - This tool can be used to adjust the contrast or brightness of displayed images
    - When used with grayscale images (scaled ΔR/R images or imported vasculature images) a new window will open in which you can interactively adjust contrast limits. When ready, click the “Adjust Data” button.
    - The adjust contrast tool is not compatible with RGB images (such as the overlay images generated by the app, see below). In this case, the image brightness will be increased iteratively by 5% each time the “Adjust Contrast” button is pressed.


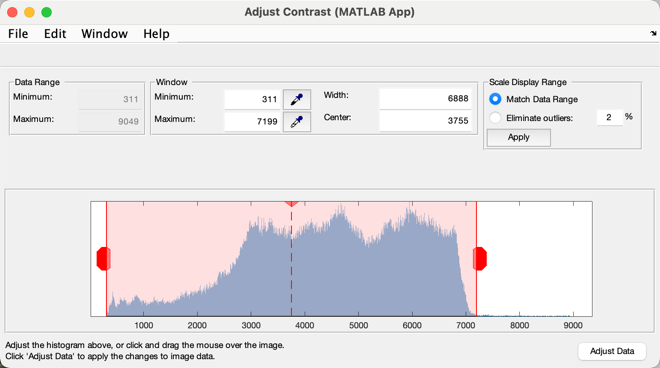


**Figure 3. The “Adjust Contrast” window.**

- - - - For publication quality RGB overlay images, we recommend using a dedicated image processing tool (such as ImageJ) to adjust the vasculature image contrast and then overlay a pseudocolored binary image. This will give the user more control over contrast and transparency then we have built into this tool.
    - Adjustments are for display purposes only and are not automatically saved. If you would like to save an image with adjusted contrast, hover over the upper right corner of the Image Axes and use the image toolbar to save manually.
- Generate Z-score Overlay
  - This button will create an image of IOSI signals overlaid onto an image of the cortical vasculature using a Z-score threshold


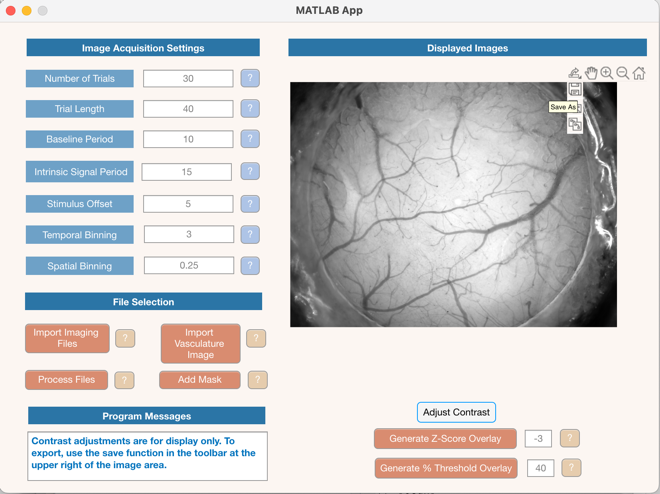


**Figure 4. The image axes toolbar.** To manually save an image, hover over the upper right corner of the axes to access the toolbar.

- - - Calculated ΔR/R values will be Z-scored and binarized according to the threshold set in the input box next to the button
      - In our experience Z-scores of -2 to -3 work well for first phase “initial dip” intrinsic signals. Smaller (more negative) Z-scores will exclude more signal, keeping just the strongest signals. Larger (more positive) Z-scores will include more signal and therefore potentially background noise. If you are measuring a later phase intrinsic signal (such as the positive-going 2^nd^ phase) these ranges will need to be adjusted accordingly.
      - Signals outside of any mask previously set will be excluded
    - The binarized signal will be pseudo-colored and overlaid onto a previously selected vasculature image. Both the binary image and the overlay will be automatically saved in the directory containing the images selected for analysis. If binary and overlay image files already exist in the directory, a timestamp will be appended to the file names to prevent overwriting of data.
- Generate % Threshold Overlay
  - This button will create an image of IOSI signals overlaid onto an image of the cortical vasculature, using a threshold set according a percentage of the total signal range
    - A threshold, based on the full range of ΔR/R values (maximum minus minimum) will be calculated according to the percentile set in the input box next to the button. The ΔR/R values will then be binarized according to the threshold, keeping only the specified percentage of the overall range of values
      - In our experience percentiles of 30-60% work well for first phase “initial dip” intrinsic signals. Smaller percentiles will exclude more signal, keeping just the strongest signals. Larger percentiles will include more signal and therefore potentially background noise. If you are measuring a later phase intrinsic signal (such as the positive-going 2^nd^ phase) these ranges will need to be adjusted accordingly.
      - Signals outside of any mask previously set will be excluded
    - The binarized signal will be pseudo-colored and overlaid onto a previously selected vasculature image. Both the binary image and the overlay will be automatically saved in the directory containing the images selected for analysis. If binary and overlay image files already exist in the directory, a time stamp will be appended to the file names to prevent overwriting of data.
