# Supplementary material for: High-Sensitivity Intrinsic Optical Signal Imaging Through Flexible, Low-Cost Adaptations of an Upright Microscope
Source: eNeuro. 2023 Aug 4;10(8):ENEURO.0046-23.2023. doi: 10.1523/ENEURO.0046-23.2023 (PMC10408783; doi:10.1523/ENEURO.0046-23.2023)
Supplement: Extended Data 1 — Extended data includes several additional files. “ISI Parts List.xlsx” is an excel spreadsheet listing all materials required to implement the IOSI adaptations described in this manuscript. “IOSI_RingLED_IlluminationBuild.docx” is a word document providing step-by-step instructions for assembling and setting up ring LED illumination. “Ring LED Holder-2.stl” is a design file to 3D print the mount for the ring LED. “NeopixelControl.mlapp” is a MATLAB application for controlling ring LED illumination. “iosgui.mlapp” is a MATLAB application for processing IOSI data. “IOSGUI_ImageAnalysis.m” is a companion script for processing IOSI data, used by “iosgui.mlapp”. “IOSGUI_Instructions.docx” is an instruction manual for using the iosgui application for processing IOSI data. Download Extended Data 1, ZIP file. [file enu-eN-OTM-0046-23-s02.zip › Extended Data/IOSI_RingLED_IlluminationBuild.docx]

**Wire the Arduino Rev 3**

1. Connect a jumper cable for output to the Neopixel to Digital Pin 6 of the Arduino Rev3
   1. Solder the other end of the output jumper cable to one wire (white) of the multi-conductor cable
2. Connect a 2^nd^ jumper cable for ground to the GND pin of the Arduino Rev3
   1. Connect the other end of the ground jumper cable to the negative screw terminal of the female DC power adapter block
3. Connect one wire (black) of the multi-conductor cable to the negative screw terminal of the female DC power adapter block
4. Connect one wire (red) of the multi-conductor cable to the positive screw terminal of the female DC power adapter block
5. Bridge the negative and positive terminals of the female DC power adapter block with a 1000 μF capacitor
6. Plug the 5V power supply into the female DC power adapter block. Do not plug the power supply into the wall into all connections have been completed.

**
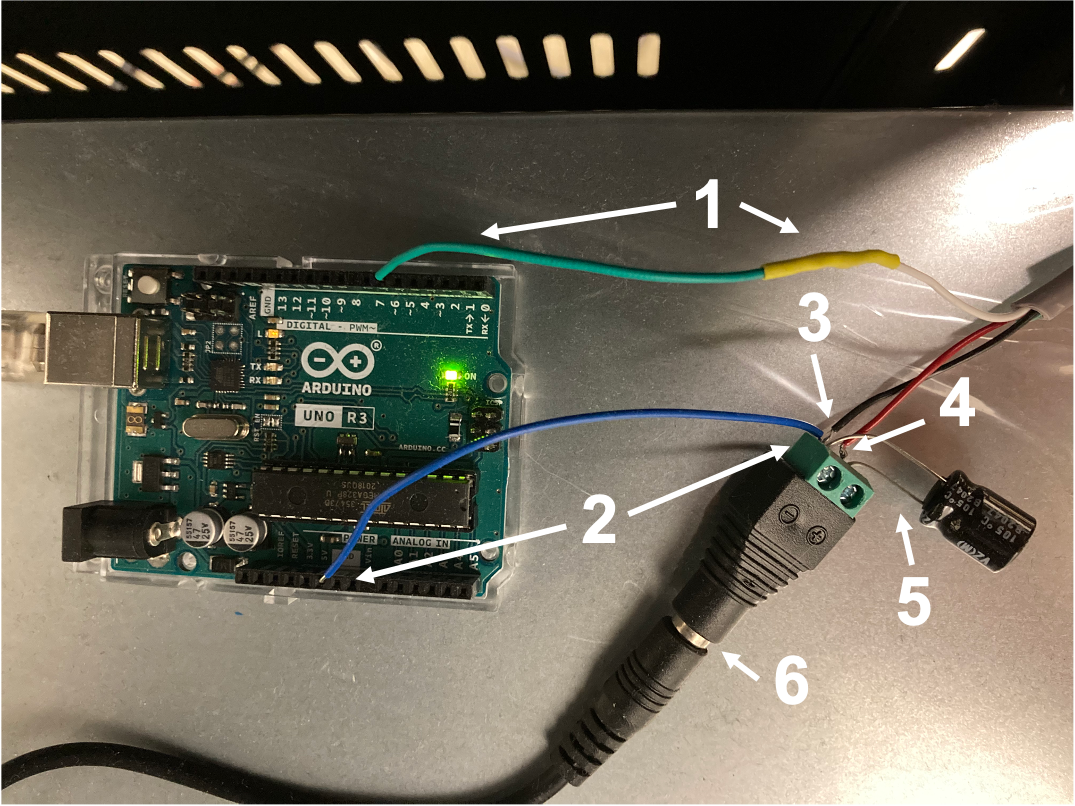
**

**Wire the Neopixel Ring**

1. Cut 3 pieces of wire (white, black, and red) to an approximate length of 5 cm
2. Solder one wire to each of 3 pins on the male connector latch holder
   1. The order does not matter, but keep track because you must match the color of each wire to its corresponding wire coming from the Arduino (see step 11 below)
3. Solder the other ends of each wire to the Neopixel Ring as follows:
   1. White to “IN” hole
   2. Black to the “G” hole (there are two G holes, either is fine)
   3. Red to the “V+” hole (there are two V+ holes, either is fine)
   4. Note: It is easiest to insert the wire from the front of the Neopixel Ring (where the LEDs are located) and solder onto the back
4. Add crimp socket connectors to the exposed ends of the multi-conductor cable (i.e. the ends NOT connected to the Arduino) of the white, black and red wires.
5. Insert the crimp socket connector covered wires into the female socket connector receptacle, ensuring that the order of wires matches the order the wires are attached to the pins of the male connector latch (see step 8 above)


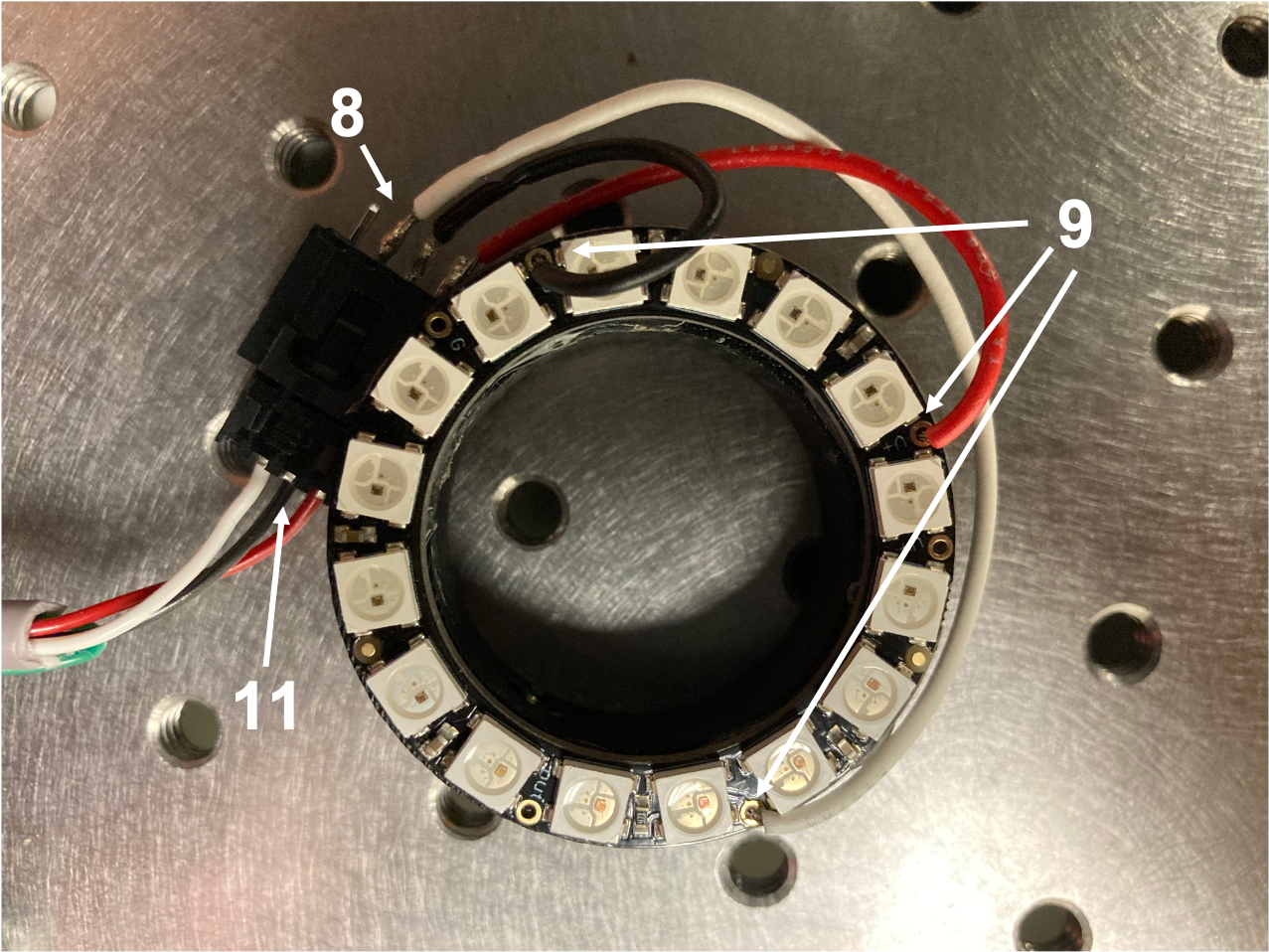


**Assemble the Neopixel Ring Holder**

1. 3D print the Neopixel Ring Holder (Ring LED Holder-2.stl)
2. Use a solder to carefully insert the heat-set 2-56 thread size screw insert through one side of the Neopixel Ring Holder
3. Once cooled, thread a 2-56 threaded nylon-tip set screw partially into the heat-set screw insert
4. Use cyanoacrylate glue (Krazy Glue, or similar) to adhere the male connector latch holder to the side of the Neopixel Ring Holder
5. Use a hot-glue gun to adhere the back side of the Neopixel Ring to the underside of the Neopixel Ring Holder
   1. Ensure that all wiring is properly soldered before adhering the Neopixel Ring to the holder, as it will be much more difficult to adjust once this step has been performed
6. Insert the female socket connector receptacle into the male connector latch holder to connect the Arduino Rev3 to the Neopixel Ring
7. Slide the assembled Neopixel Ring Holder onto the 4x microscope objective. Tighten the set-screw gently to ensure the Neopixel Ring Holder will not fall off


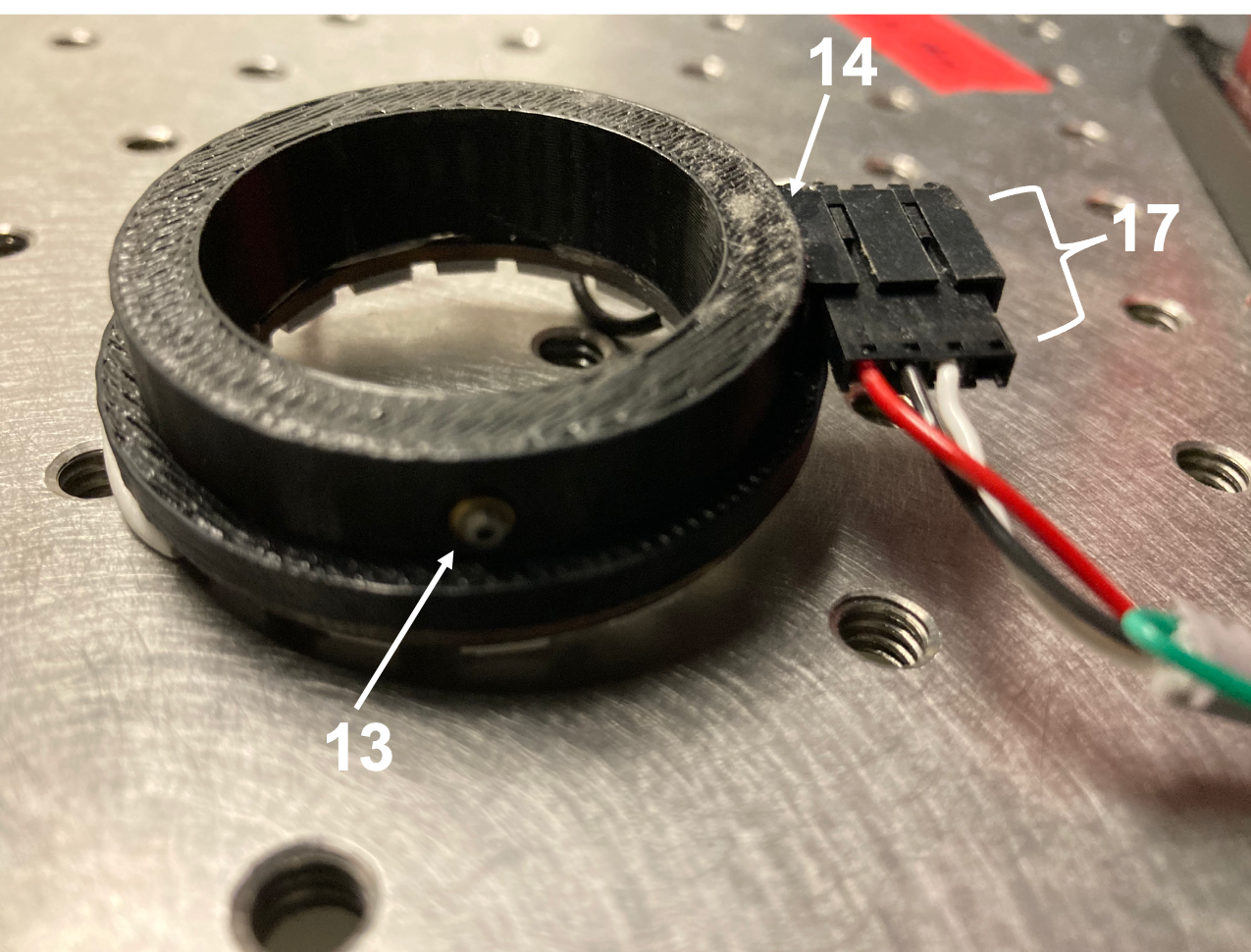


**Install Software**

1. Plug in the 5V power supply
2. Connect a USB Type A/B cable between a computer and the Arduino Rev3
3. Download and install the Arduino IDE (<https://www.arduino.cc/en/software>)
4. Download and install the Adafruit Neopixel Library (https://github.com/adafruit/Adafruit_NeoPixel)
5. Download and install MATLAB (<https://matlab.mathworks.com>)
6. Download and install the MATLAB Arduino Support Package (https://www.mathworks.com/hardware-support/arduino-matlab.html)
7. Download and install the MATLAB Neopixel Add-On Library for Arduino (https://www.mathworks.com/matlabcentral/fileexchange/72707-neopixel-add-on-library-for-arduino)
8. Clone our intrinsic signal imaging repository and add to your MATLAB path [URL redacted for double-blind review]
9. Determine which port your Arduino is connected to. You may need to change a line of the NeopixelControl.mlapp code to ensure this is correct
   1. Open “Device Manager” in Windows and expand the “Ports (COM & LPT)” list (https://www.mathworks.com/help/supportpkg/arduinoio/ug/find-arduino-port-on-windows-mac-and-linux.html)
      1. Find the Arduino Uno and make note of the port listed (e.g. “COM5”)
   2. Adjust the first input variable in line 31. For example:
      1. a = arduino('COM5', 'Uno', 'Libraries', 'Adafruit/NeoPixel');
   3. If you have adapted this protocol to use a different Neopixel Ring, you may need to further adjust the code to match the specifications of your Neopixel Ring
10. From the MATLAB command window, type NeopixelControl to open the app to control the Neopixel Ring illumination
